# Efficacy of acupuncture for chronic migraine: a protocol for a multicenter randomized controlled trial

**DOI:** 10.3389/fneur.2025.1647966

**Published:** 2025-12-24

**Authors:** Qianxiu Chen, Zhe Wang, Yu Tang, Xinyu Wu, Yijie Wang, Tianyu Bai, Ying Gao, Huafeng Cui, Jiamei Zhang, Pengcheng Li, Jing Han

**Affiliations:** 1 College of Acupuncture and Tuina, Shandong University of Traditional Chinese Medicine, Jinan, Shandong, China; 2 Department of Acupuncture, Yantai Traditional Chinese Medicine Hospital, Yantai, Shandong, China; 3 Department of Acupuncture, Shandong Provincial Third Hospital, Jinan, Shandong, China; 4 Department of Acupuncture, First Teaching Hospital of Tianjin University of Traditional Chinese Medicine, Tianjin, China; 5 Department of Acupuncture, Affiliated Hospital of Shandong University of Traditional Chinese Medicine, Jinan, Shandong, China

**Keywords:** acupuncture, chronic migraine, protocol, RCT, trial

## Abstract

**Introduction:**

Chronic migraine (CM) is a prevalent neurovascular disorder that significantly affects quality of life. Research has shown promising results for the efficacy of acupuncture in the treatment of chronic migraine. This study aims to evaluate the efficacy of acupuncture as a prophylactic treatment for chronic migraine through a multicenter randomized controlled trial.

**Materials and methods:**

This investigation constitutes a prospective, two-arm, multicenter randomized controlled trial involving a total of 180 patients diagnosed with chronic migraine (CM). Participants will be allocated in a 2:1 ratio to either the treatment group, receiving acupuncture, or the control group, receiving sham acupuncture. The intervention will be administered three times per week over a 6-week treatment period, followed by an 8-week follow-up phase. The primary outcome measure is the change from baseline in the average number of monthly headache days during weeks 3–6 of the treatment period. Secondary outcomes include changes in monthly headache days during follow-up, acute medication use days (AMDs), Migraine-Specific Quality of Life (MSQ), headache symptom scores, and pain severity as assessed using the visual analog scale (VAS).

**Ethics and dissemination:**

The study was approved by the Ethics Committee of Shandong University of Traditional Chinese Medicine (approval number: 2024-014-01-KY) and is registered with the Chinese Clinical Trial Registry (registration number: ChiCTR2400084720). Written informed consent was obtained from all participants. The results will be disseminated through peer-reviewed publications and conference presentations.

**Clinical trial registration:**

https://www.chictr.org.cn/showproj.html?proj=226947, identifier ChiCTR2400084720.

## Introduction

Migraine is a neurovascular disorder that affects approximately 15.2% of the global population. It ranks as the second leading cause of health loss in terms of years lived with disability worldwide and is the leading cause among women aged 15–49 years (1). Chronic migraine (CM), defined by the International Classification of Headache Disorders, 3rd edition (ICHD-3), as experiencing headaches occurring on ≥15 days per month for >3 months with at least 8 days per month fulfilling criteria for migraine, represents the most severe form of migraine and affects approximately 1–3% of the general population (2). Compared to those with episodic migraine, patients with chronic migraine have a higher prevalence of depression and anxiety, dyspepsia, irritable bowel syndrome, epilepsy, chronic sinusitis, and many other disorders (3). Current pharmacological treatments for CM include oral medications such as topiramate, valproate, propranolol, and injectable botulinum toxin A (BoNT-A), as well as recently developed monoclonal antibodies targeting the calcitonin gene-related peptide (CGRP) pathway (4, 5). However, these pharmacological treatments may cause adverse events (AEs) such as hypotension, depression, somnolence, and gastrointestinal intolerance (6). These comorbidities and adverse events not only impose a greater burden on patients but also significantly impair their health-related quality of life, negatively affecting treatment response, medication adherence, and overall wellbeing (7). Therefore, the effective management of chronic migraine requires a comprehensive strategy that not only focuses on ameliorating headache symptoms but also prioritizes improving medication adherence, emphasizing daily disease management, and ultimately enhancing the patient’s overall quality of life.

Acupuncture, a non-pharmacological therapy with a long history in Traditional Chinese Medicine, is increasingly recognized globally for its favorable safety profile and low incidence of adverse events (8, 9). Multiple high-quality systematic reviews (10, 11) and randomized controlled trials (RCTs) (12, 13) have consistently established the effectiveness of acupuncture as a prophylactic treatment for migraine, demonstrating significant long-term reductions in headache frequency and severity in the general migraine population. However, the majority of existing high-quality clinical studies have primarily focused on patients with episodic migraine, while dedicated research targeting patients with chronic migraine (CM), who experience more severe symptoms and greater management complexity, remains relatively limited (14). CM patients experience a significantly higher disease burden compared to EM patients (15), and the effective management of CM should adopt a comprehensive strategy that not only alleviates pain symptoms but also prioritizes improving medication adherence, managing the associated emotional burden, and ultimately enhancing the patient’s overall health-related quality of life (16).

Therefore, the present study is designed to rigorously evaluate acupuncture as a prophylactic treatment for chronic migraine using a multicenter randomized controlled trial. It is hypothesized that patients who receive acupuncture will demonstrate significantly greater reductions in monthly headache days, improved migraine-specific quality of life, and reduced acute medication use compared to those who receive sham acupuncture.

## Materials and methods

### Study design

**Figure 1:**
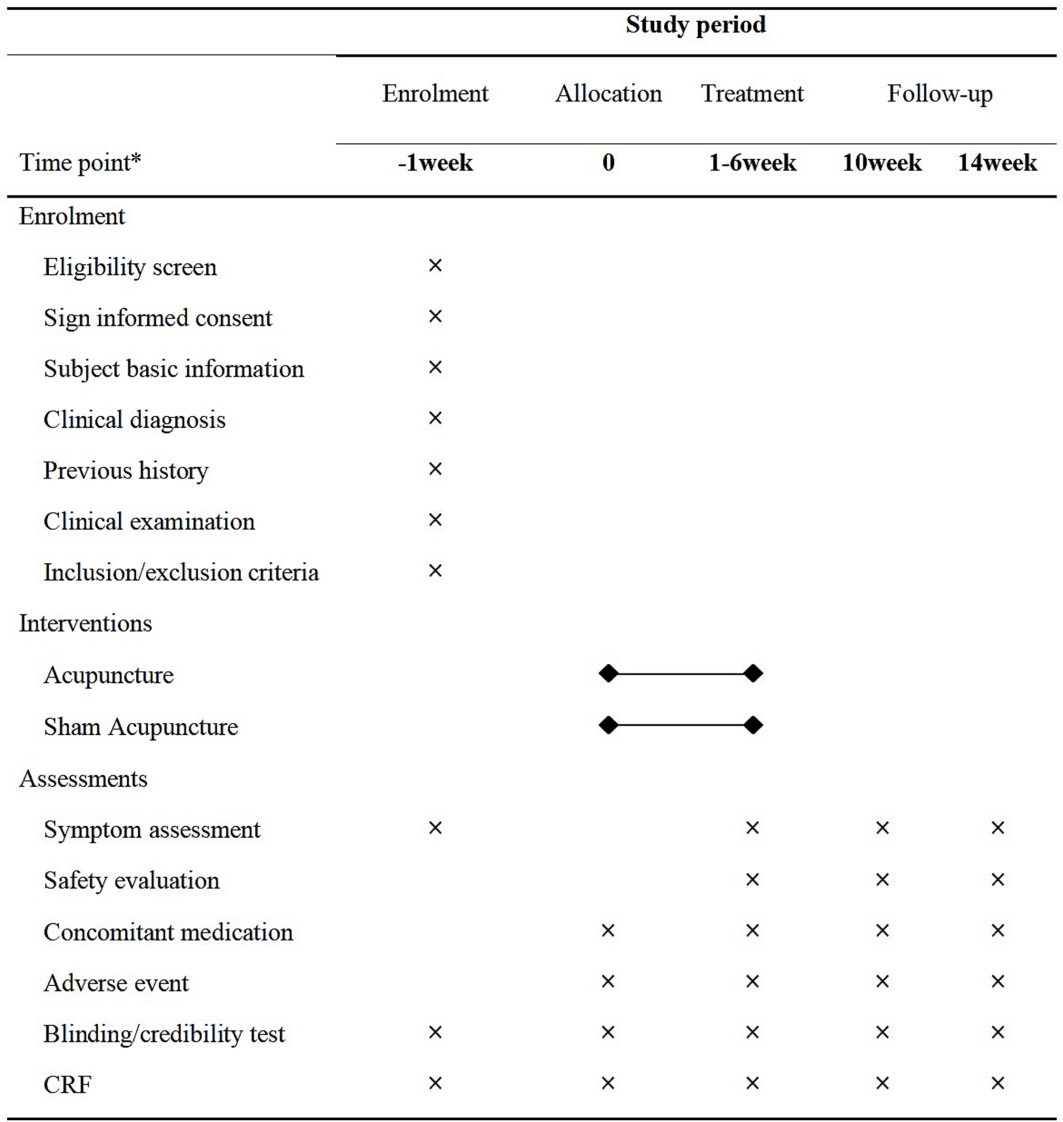
Timeline for all patients to complete assessments.

**Figure 2:**
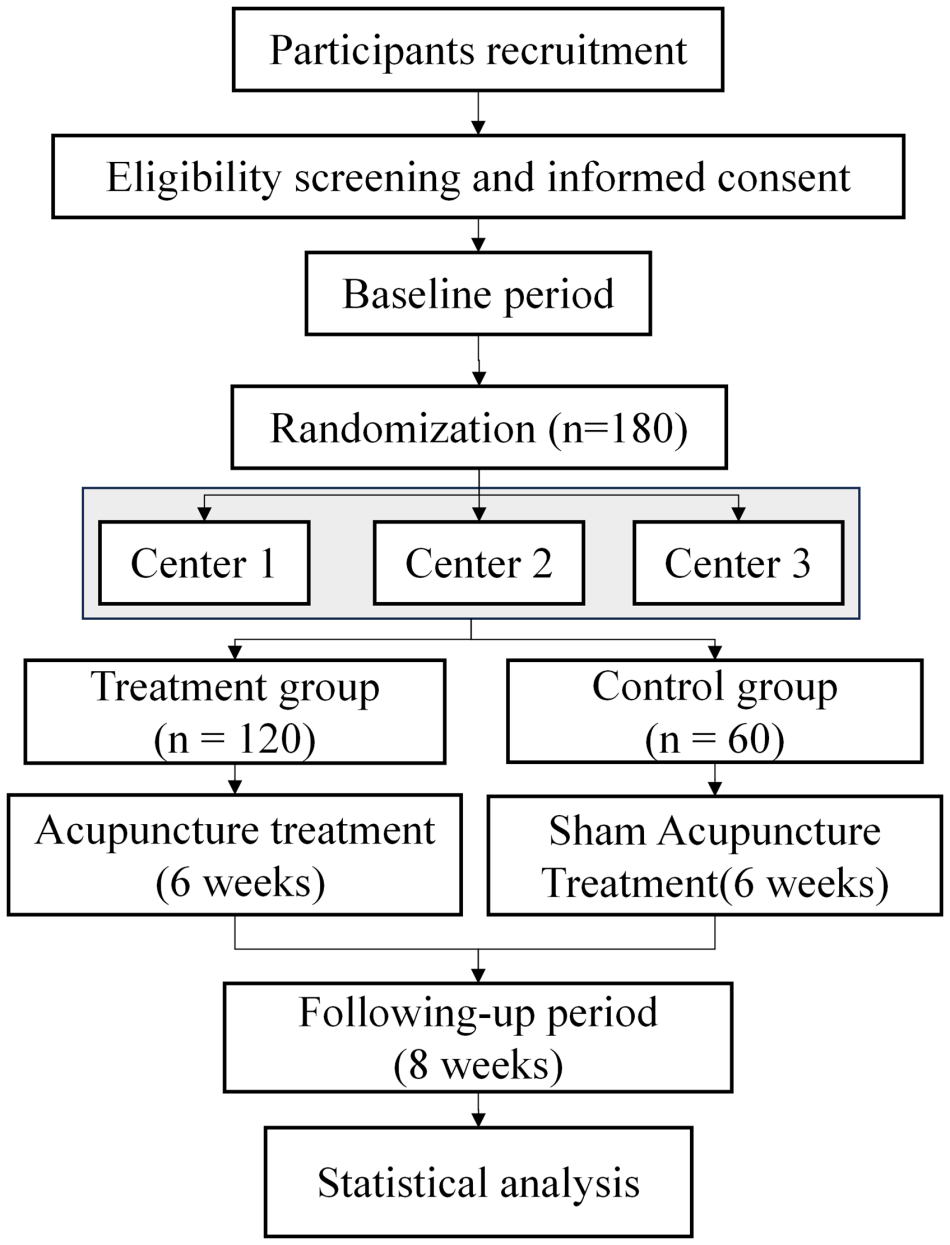
Study flowchart.

This study is a prospective, two-arm, multicenter, randomized controlled trial. A total of 180 patients with chronic migraine will be allocated in a 2:1 ratio to a treatment or control group. The trial will consist of 6 weeks of acupuncture or sham acupuncture treatment, followed by an 8-week follow-up period. The study centers are the Affiliated Hospital of Shandong University of Traditional Chinese Medicine, Yantai Traditional Chinese Medicine Hospital, and Shandong Provincial Third Hospital. Patient recruitment has been ongoing since May 2024, and the timeline for all patients to complete the assessment is shown in Figure 1. The study flowchart is shown in Figure 2. The protocol adheres to the principles of the Declaration of Helsinki and will be reported in accordance with the Standard Protocol Items: Recommendations for Interventional Trials guidelines (Supplementary File S1).

### Recruitment

Participants are being recruited from individuals diagnosed with chronic migraine according to the criteria established by the International Headache Society (IHS). Recruitment is taking place across the three previously designated medical centers. Recruitment advertisements have been published through posters and social media to attract potential participants. Clinicians will determine in person whether patients meet the strict inclusion and exclusion criteria for the study. Patients who meet the criteria and volunteer to participate will sign a written informed consent form before the start of the study. They will be fully informed of the study details, including procedures, potential benefits, and risks, with the exception of specific information regarding the needling site. Participants will be free to withdraw from the study at any time without any negative impact on their future medical treatment. In addition, measures will be taken to ensure the confidentiality of participants’ data.

### Inclusion criteria

1. Patients who meet the diagnostic criteria for chronic migraine according to the 2018 International Classification of Headache Disorders (3rd edition) (ICHD-3) (2).
2. Patients aged 18–65 years.
3. Patients who have not received acupuncture treatment in the past 3 months;
4. Patients who voluntarily agree to participate and sign the informed consent form.

### Exclusion criteria

1. Individuals prone to concurrent infections and bleeding, lactating women, pregnant women, and individuals with allergies (especially those allergic to metal needles).
2. Individuals with serious systemic diseases, such as heart, liver, kidney, or brain disorders.
3. Individuals with mental or somatic disorders who are unable to independently complete the clinical study.
4. Individuals who have taken migraine prevention drugs, anti-anxiety medications, or antidepressants within the past month of the study.
5. Individuals who are unable to cooperate with the treatment and complete follow-up or those who are currently participating in other relevant clinical trials.

### Randomization and allocation concealment

Participants in this study are strictly being screened according to the diagnostic, inclusion, and exclusion criteria. A random number list was generated using SPSS 25.0 software and converted into random number cards. Participants will be randomly allocated into two groups in a 2:1 ratio: 120 participants in the acupuncture treatment group and 60 of them in the control group. The allocation process will be kept confidential using sealed, opaque envelopes. The random number cards will be placed inside these envelopes, which will be numbered sequentially. The envelopes will be opened in the order in which the participants enter the study, and the participants will be assigned to their respective groups based on the number inside the envelope, ensuring a 2:1 allocation ratio between the groups.

### Blinding

Due to the specific nature of acupuncture, it is not feasible to blind practitioners. Therefore, blinding will be implemented for the participants, evaluators, and statisticians. Practitioners adhere to strict confidentiality protocols to avoid disclosing group allocation to the participants and evaluators. Evaluators are responsible for collecting and analyzing the data unrelated to the treatment process and are not exposed to any information about group allocation throughout the study. Statisticians are solely responsible for data analysis and are not involved in the treatment process, thereby ensuring the objectivity and impartiality of the results.

To evaluate participant blinding integrity, Bang’s Blinding Index (BI) will be calculated at the completion of the treatment phase (17). After the final treatment session, the participants will be asked to guess their group allocation (real acupuncture, sham acupuncture, or unsure) and to rate their confidence in this guess.

The Credibility/Expectancy Questionnaire (CEQ) (18) will be administered after the first treatment session. Group differences in the CEQ scores will be examined to verify that expectancy was balanced between the groups.

After the completion of statistical analysis, the unblinding procedure will be initiated. Researchers responsible for unblinding will disclose group information to the data analysts as necessary to finalize comparisons and interpret the study results.

### Interventions

The intervention measures will be in accordance with the Uniform Standard for Trial Reporting (19) and the Standard for Reporting Interventions in Clinical Trials of Acupuncture and Moxibustion (20).

#### Treatment group

##### Acupoint selection

**Figure 3:**
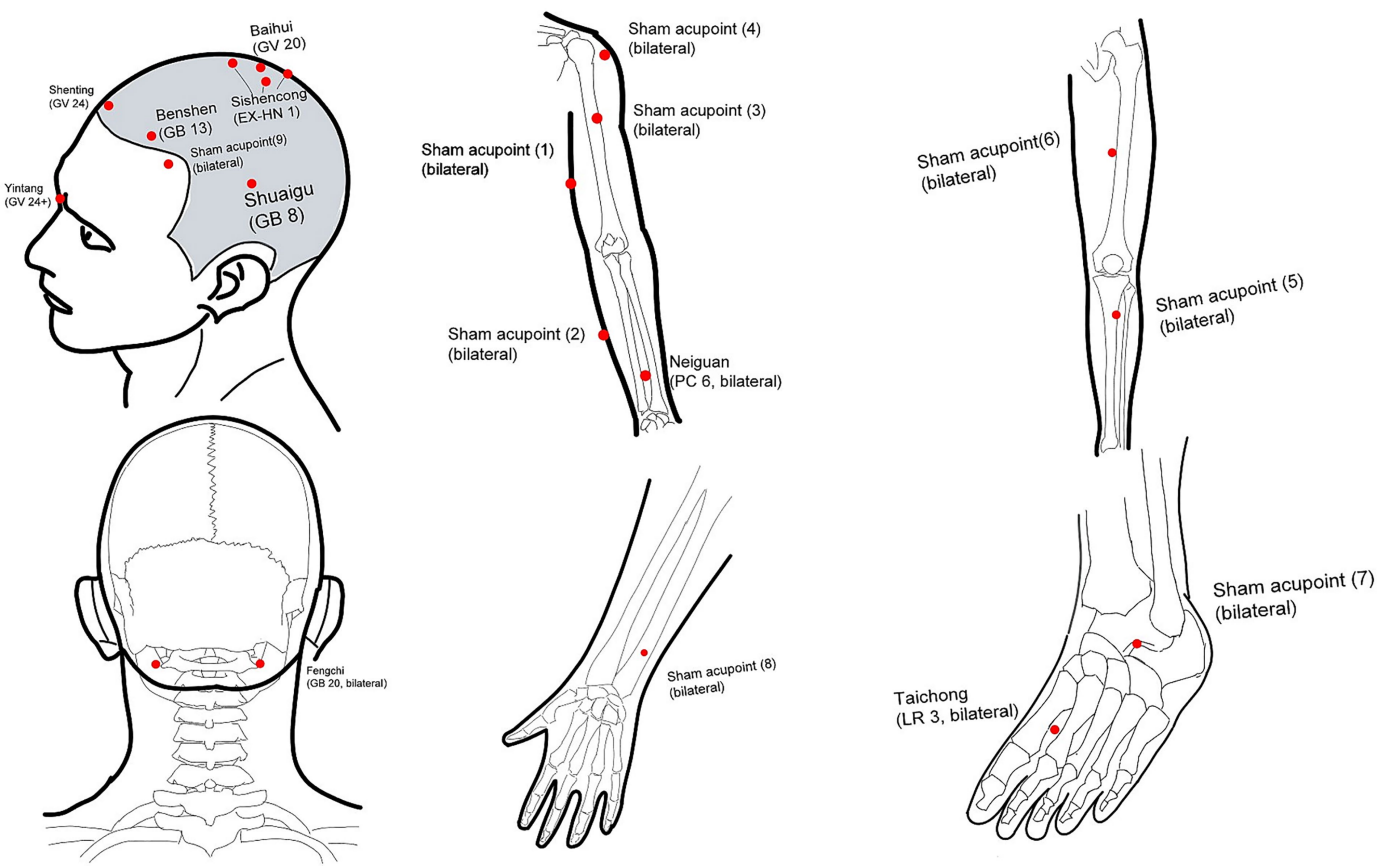
Location of acupoints and sham acupoints.

Based on clinical experience and previous research (21–24), and by incorporating suggestions from consensus meetings with clinical experts, the following acupoints will be selected: Baihui (GV20), Yintang (GV24+), Sishencong (EX-HN1), Shenting (GV24), Benshen (GB13, bilateral), Shuigu (GB8, bilateral), Fengchi (GB20, bilateral), Neiguan (PC6, bilateral), and Taichong (LR3, bilateral) (Table 1 and Figure 3).

**Table 1:** Acupoints and description of acupoint locations.

Location of real acupoints used in the real acupuncture treatment group	
Acupoints	Location
Baihui [GV20]	On the head, 5 cun directly above the anterior hairline.
Yintang (GV24+)	On the head, in the depression at the midpoint of the inner ends of the eyebrows.
Sishencong (EX-HN1)	On the top of the head, 1 cun anterior, posterior, and lateral to Baihui, forming a total of four points.
Shenting (GV24)	On the head, 0.5 cun directly above the anterior hairline.
Benshen (GB13)	On the head, 0.5 cun above the anterior hairline, 3 cun lateral to the midline of the head.
Shuaigu (GB8)	On the head, 1.5 cun directly above the apex of the ear.
Fengchi (GB20)	On the posterior neck area, in the depression below the occipital bone, between the upper ends of the sternocleidomastoid and trapezius muscles.
Neiguan (PC6)	On the anterior forearm, 2 cun proximal to the wrist crease on the palmar side, between the palmaris longus tendon and the radial flexor carpi tendon.
Taichong (LR3)	On the dorsum of the foot, in the depression between the 1st and 2nd metatarsal bones, or where the pulse can be felt.

Location of sham acupoints used in the sham acupuncture control group	
Sham acupoints	Location
Sham acupoint (1)	On the inside of the elbow, at the midpoint of the line connecting the tip of the elbow and the armpit.
Sham acupoint (2)	On the ulnar side of the forearm, at the midpoint of the line connecting the medial wrist crease and the medial epicondyle of the humerus.
Sham acupoint (3)	At the anterior border of the arm, at the junction between the deltoid and biceps muscles.
Sham acupoint (4)	At the midpoint of the line connecting the deltoid muscle and the acromion.
Sham acupoint (5)	1–2 cm lateral to the Zusanli (ST 36) point, along the outer edge of the tibia.
Sham acupoint (6)	On the rectus femoris muscle, at the midpoint of the line connecting the anterior superior iliac spine and the lateral end of the patella, 2 cm medial.
Sham acupoint (7)	At the midpoint of the line connecting the Qiuxu (GB40) and Jiexi (ST41) points.
Sham acupoint (8)	Between the Sanjiao Meridian of Hand-Shaoyang and the Small Intestine Meridian of Hand-Taiyang, at the level of Waiguan (TE 5), pointing outward.
Sham acupoint (9)	0.5 cun below the hairline at the corner of the forehead, 4 cun lateral to the midline (below and in front of the Touwei (ST 8) point).

##### Acupoint location

The acupoint locations were referenced from the 2010 WHO standard (ISBN: Tel: 9787117123327). The acupoint locations are shown in Table 1 and Figure 3.

##### Material preparation

Needles: Huatuo brand disposable sterile acupuncture needles, produced by Suzhou Medical Supplies Factory Co., Ltd., with specifications of Φ0.30 mm × 40 mm, will be used.

Other Materials: 75% alcohol for disinfection and sterile dry cotton swabs will be used.

##### Operation method

The acupuncture treatment will follow a standardized procedure. The operator will disinfect their hands. The patient will first sit up, and the Fengchi point (GB 20) will be disinfected. The operator will then insert the needle at a depth of 0.8–1.2 cun, angled toward the tip of the nose at Fengchi (GB20), and will withdraw it after achieving Deqi (a sensation that may include soreness, numbness, distention, or heaviness). The patient will then lie supine, and the acupoints Baihui (GV20), Sishencong (EX-HN1), Benshen (GB13), and Shenting (GV24) will be disinfected and needled at a depth of 0.5–0.8 cun. Yintang (GV24+) will be needled downward at a depth of 0.5–1 cun; Shuigu (GB8) at a depth of 0.5–0.8 cun; and Neiguan (PC6) and Taichong (LR3) at a depth of 0.5–0.8 cun. All needling points will be required to achieve Deqi, and after achieving Deqi, the needles will be retained for 30 min.

A “cun” is a proportional body measurement unit used in acupuncture, defined relative to each patient’s anatomical landmarks. For example, the distance between the two ends of the skin crease of the interphalangeal joint of the patient’s middle finger, when flexed, is considered 1 cun. Deqi (obtaining qi) refers to a composite of needle sensations, including soreness, numbness, heaviness, and distension, which indicate effective acupuncture stimulation and are considered important for the therapeutic response. In this trial, deqi will be assessed by practitioners through tactile feedback (needle grasp and tissue response) and patient-reported sensations after gentle needle manipulation.

##### Treatment course

The treatment will be administered every other day, three times per week, for a total of 6 weeks. During the treatment course, symptom assessment and clinical safety evaluation will be conducted as needed, monitoring for adverse events, recording concomitant medications, and documenting protocol deviations. Case report forms (CRFs) will be completed.

#### Control group

The sham acupoint locations that will be employed in this trial have been selected based on validated protocols from previous migraine acupuncture RCTs (25–27). These non-acupoint positions meet three critical design criteria: (1) anatomical validity—located outside traditional meridian pathways to minimize point-specific activation; (2) sensory similarity—positioned to provide comparable tactile sensations for maintaining participant blinding; and (3) established precedent—these sham points have been validated across multiple high-quality migraine trials, including large-scale studies demonstrating successful group differentiation. The control group will receive sham acupuncture at the designated Sham acupoints.

All Sham acupoints will be shallowly needled bilaterally at a depth of approximately 2 mm. No needling manipulation will be performed, no qi will be obtained, and needles will be retained for 30 min. Needle specifications and the treatment course will be the same as those in the treatment group (Table 1 and Figure 3).

#### Concomitant treatment and follow-up

During the 8-week follow-up period, symptom assessment and clinical safety evaluation will be performed, with monitoring for adverse events, recording protocol deviations, and completing CRFs. During the study, no additional medications will be used by the participants. If pain due to migraine necessitates the use of painkillers, each instance of medication use must be reported to the researcher, with detailed records of the medication name, specifications, dosage, time of use, and symptom relief documented.

### Outcome measures

#### Primary outcome measures

The primary outcome measure is the change from baseline in the average number of monthly headache days during weeks 3–6 of the 6-week treatment period.

According to the headache diary, patients will be asked about the duration of headache, defined as the number of days on which the headache lasted for at least 4 h. Headaches will be considered according to the ICHD-III criteria: criteria C and D (1.1) for migraine without aura, criteria B and C (1.2) for migraine with aura, or criteria 1.6 for possible migraines. Patients will also be required to record, on days with a migraine or headache, successful treatment with trastum, ergot, or other migraine-specific acute drugs.

#### Secondary outcome measures

Secondary outcome measures include the following:

Changes from baseline in the average number of monthly headache days assessed during the 8-week follow-up period, specifically at two time windows: weeks 7–10 (Follow-up weeks 1–4) and weeks 11–14 (Follow-up weeks 5–8).

Changes from baseline in the average monthly acute medication use days (AMDs) during the treatment period and at follow-up time points. AMD is defined as the number of calendar days per month on which the participants used any acute migraine medication. AMD will be assessed at baseline, during the treatment period (weeks 3–6), and at follow-up time points (weeks 10 and 14).

The degree of pain will be evaluated using the visual analog scale (VAS).

The migraine-specific quality of life will be measured using the Migraine-Specific Quality of Life Questionnaire(MSQ) (28) from baseline to endpoints.

The headache symptom score will be evaluated based on the standards outlined in the “Guiding Principles for Clinical Research of New Chinese Medicines,” issued by the Ministry of Health in 2002, to assess various indicators.

### Safety monitoring and adverse event reporting

To maximize participant safety and proactively minimize risks, all participants will undergo a careful anamnesis and baseline assessment before the first treatment. During the study, the participants undergoing acupuncture treatment may experience adverse events (AEs), including but not limited to pain, subcutaneous congestion, hematoma, local infection, and, in rare cases, severe events such as syncope. Safety assessments will encompass vital sign measurements (blood pressure, heart rate, respiration rate, body temperature) and physical examinations. These vital sign measurements will be performed before the start of the protocol (at baseline assessment) and at key points during the treatment period (before and after treatment). Their primary purpose will be to monitor participant safety and ensure early detection of potential adverse reactions, rather than to serve as clinical indicators of acupuncture efficacy. Detailed documentation of AEs will include the time of occurrence, duration, classification, severity, remedial actions, and final resolution. Participants will be instructed to report any abnormal reactions or discomfort, which will be carefully documented, including the time, severity, and potential causes. Regardless of their relationship to the treatment, all AEs will be promptly and appropriately managed, recorded in the Case Report Forms (CRFs), and reported to the study director and the Ethics Committee of the Affiliated Hospital of Shandong University of Traditional Chinese Medicine.

Mild and moderate AEs will be managed symptomatically and monitored, while severe AEs, such as death, life-threatening disability, or hospitalization, will receive emergency treatment and will be reported to the Research Ethics Committee within 24 h, which will provide guidance on whether the trial should be terminated. To minimize the risk of severe adverse events, all practitioners have received specific training on recognizing and managing autonomic adverse responses to acupuncture, consistent with safety guidelines outlined in recent systematic reviews (29, 30).

### Sample size

The sample size was calculated using the PASS 11.0 software. Based on published literature (12), the number of migraine attack days was estimated as the outcome indicator for long-term efficacy observation. The number of migraine attack days in the acupuncture treatment group was reduced by 3.9 ± 3.0 days, while the number of migraine attack days in the sham acupuncture control group was reduced by 2.2 ± 3.2 days. The level of statistical significance was set at *α* = 0.05, and test efficacy was defined as (1-*β*) = 0.90. The ratio of the sample size between the treatment and control groups was set at 2:1, resulting in an estimated requirement of 107 participants in the treatment group and 54 in the control group. Considering potential dropout during the trial, with a predicted shedding rate of 10%, the initial number of participants was set at 120 in the treatment group and 60 in the control group, totaling 180 participants.

### Data management

All personnel involved in data management, including data collectors, data managers, and statisticians, will receive training to ensure adherence to the study’s data management protocols. Each participant’s CRF will be meticulously completed by the investigator based on the participant’s hospital records and original observation notes. Data entry will be conducted using the EpiData 3.1 software with a double-entry system by independent data entry personnel. The data manager, in collaboration with the principal investigator, will define the range and logical checks for the data and program these into the system to prevent errors. Any discrepancies found during data entry or validation will be documented and corrected, with records of the errors and corrections maintained.

All data, including paper CRFs and electronic files, will be securely stored. Paper records will be archived in a designated order and stored in a secure location, and electronic documents will be securely stored on a passwordprotected computer. These records will be preserved for at least 5 years following publication, in accordance with national regulations on clinical trial data management.

The Ethics Committee will periodically review the trial’s progress, monitoring data collection, allocation, and security. A formal data monitoring committee (DMC) will not be established. This decision is justified by the low-risk nature of the interventions (acupuncture and sham acupuncture) when performed by certified practitioners, the relatively short duration of the treatment phase (6 weeks), and the fact that no formal interim analyses for efficacy are planned. Instead, safety monitoring responsibilities will be jointly handled by the principal investigators and the Ethics Committee, which will conduct periodic reviews of safety data (including all AEs and SAEs) in addition to its standard oversight of trial conduct.

### Data analysis

Statistical analysis will be performed using SPSS 25.0 and the R software package. The primary analysis will be based on the full analysis set (FAS), which will include all participants who receive at least one acupuncture treatment and have primary outcome data. Missing baseline data for primary outcomes will lead to exclusion from the FAS. Sensitivity analysis will be conducted using the per-protocol set (PPS), comprising participants who meet all inclusion and exclusion criteria, have valid baseline data, demonstrate good compliance, and have not violated the study protocol. Safety analysis will be performed based on the safety set (SS), which will include all participants who receive at least one acupuncture treatment.

Descriptive statistics will be used to summarize demographic and baseline characteristics. Categorical data will be presented as counts (n) and percentages (%), with differences between the groups analyzed using the chi-squared test or Fisher’s exact test. Continuous variables will undergo normality testing. Normally distributed data will be expressed as mean±standard deviation ($\bar{X}$ ± S) and compared between the groups using independent samples *t*-tests. Non-normally distributed data will be presented as medians and interquartile ranges [M, (P25, P75)] and analyzed using the Mann–Whitney U test. Repeated measures data that meet the assumptions of normality, homogeneity of variance, and sphericity will be analyzed using repeated measures ANOVA, with corrections applied as necessary for violations of sphericity. To ensure the study’s scientific reliability and rigor, we will work to keep missing data under 20% and will use multiple imputation to handle missing data for the primary outcome.

Bang’s Blinding Index will be calculated for each group with 95% confidence intervals using the Wilson score method. Blinding will be considered successful if BI values are close to zero (|BI| < 0.3). Group differences in the CEQ scores will be compared using independent samples *t*-tests (or the Mann–Whitney U test if data are non-normally distributed). If the CEQ scores differ significantly between the groups, sensitivity analyses will include the CEQ as a covariate in primary outcome models (ANCOVA or mixed models) to adjust for potential expectancy effects. Correlation analyses will explore associations between the CEQ scores, acupuncture sensation scores, and primary outcomes to identify potential mediators of treatment effects.

Safety analysis will include the incidence of adverse events (AEs) during the study, with group comparisons of treatment-related adverse events conducted using a chi-squared test or Fisher’s exact test. Additional analyses may be conducted as deemed appropriate, with further details provided in the statistical analysis plan. Statistical significance will be set at a *p*-value of <0.05 for all analyses.

### Quality control

The acupuncture procedure will primarily be carried out by acupuncture specialists with more than 10 years of clinical experience. A launch meeting has been convened before the formal initiation of the trial to provide training on the purpose and design of the trial, treatment strategies, and quality control for all staff. The clinical participants will be required to complete the CRFs according to the design requirements, filling them in carefully and in detail. The study medical records and the CRFs shall be the original records and shall not be altered. All data in the clinical trial should be recorded, and the original report (or copy) should be attached to the case report form. Data that are significantly high or outside the clinically acceptable range will be verified, and necessary explanations will be provided by the physicians participating in the clinical trial. Clinical supervisors make regular on-site visits to research centers, check original information on a sample basis, hold regular meetings on specific issues, and provide timely feedback in the form of supervision reports and subject briefings to improve the quality of supervision.

### Patient and public involvement

Patients and the public are not involved in the planning, design, recruitment, or conduct of the study. The results of the study will be disseminated to the participants and the broader public through various channels, including educational talks, booklets, and publication in open-access peer-reviewed journals.

### Ethics and dissemination

This study was approved by the Ethics Committee of Shandong University of Traditional Chinese Medicine on 6 March 2024 (2024–014-01-KY) and conforms to the principles of the Declaration of Helsinki. The trial is registered in the Chinese Clinical Trial Registry (Registration No: ChiCTR2400084720; Register date: May 23, 2024). Written informed consent will be obtained from all participants prior to their inclusion in the trial. Data will be handled anonymously, and only participant codes will be available in the central database to protect confidentiality. All personal information outside the scope of this trial will not be collected, shared, or maintained. Participants retain the right to withdraw from the study at any time. The results of this study will be disseminated through publications in peer-reviewed journals and presentations at academic conferences.

### Trial status

Recruitment for this study began on 23 May 2024 and is scheduled to continue until 31 December 2026. The trial is currently in the participant recruitment phase.

## Discussion

First, given the inherent nature of acupuncture interventions, blinding of the treating practitioner is unfeasible, which may introduce bias. However, throughout the trial, different researchers will oversee random allocation, patient recruitment, acupuncture treatment, outcome assessment, and data analysis to mitigate bias.

Second, this trial does not include an active pharmacologic control group, such as topiramate, propranolol, or CGRP monoclonal antibodies, which represents a significant limitation. While this trial is designed to assess whether acupuncture is superior to sham acupuncture in reducing monthly headache days, the magnitude of its benefit relative to established guideline-recommended drug treatments will remain uncertain. This will limit our ability to definitively position acupuncture within the treatment algorithm for chronic migraine prevention.

However, this limitation should be interpreted in context. The primary objective of this exploratory trial is to establish whether acupuncture produces specific effects beyond placebo—a necessary first step before investing in large-scale comparative effectiveness trials. Prior network meta-analyses have suggested that acupuncture demonstrates efficacy comparable to topiramate and other first-line preventives for chronic migraine (31), and recent head-to-head trials have shown non-inferior efficacy with superior tolerability (32). Nonetheless, future multi-arm trials directly comparing acupuncture, sham acupuncture, and active drug controls are warranted to provide definitive evidence for clinical guideline development, insurance coverage decisions, and shared decision-making between patients and clinicians regarding optimal treatment selection.

Third, the study employs a relatively short 8-week post-treatment follow-up period, which limits the assessment of long-term efficacy and durability. Migraine prevention guidelines often recommend 3–6 month follow-up to comprehensively assess sustained effects and potential relapse rates, which our 8-week window cannot capture. While this timeframe is shorter than in some prominent acupuncture and pharmacologic trials, it represents a scientifically sound and pragmatically feasible design for an exploratory study focused on short-term prophylactic efficacy. Therefore, should this trial demonstrate efficacy, future confirmatory studies with extended follow-up (e.g., 6–12 months) will be essential to fully characterize acupuncture’s long-term potential, identify relapse patterns, and establish evidence-based maintenance recommendations.

Chronic migraine (CM) is a neurovascular disorder that imposes a significant burden on individuals and healthcare systems globally (15, 33). Despite the availability of pharmacological treatments, including nonsteroidal anti-inflammatory drugs (NSAIDs), opioids, and triptans (4), these interventions present several limitations (34). Many patients report inadequate pain relief, frequent recurrence, and adverse effects, such as gastrointestinal disturbances and medication overuse headaches (9), which undermine long-term compliance and efficacy (35).

As a non-pharmacological intervention, acupuncture is associated with fewer side effects and has shown potential in reducing headache frequency, intensity, and duration (36–38). Moreover, it may alleviate associated symptoms and enhance overall wellbeing (32).

This study aims to provide recent high-quality evidence on the clinical efficacy of acupuncture for chronic migraine (CM). This randomized controlled trial will offer detailed and clinically relevant insights into the mechanisms and effects of acupuncture in CM management.
